# Social support and aging: psychometric analysis of the ENRICHD Social Support Instrument in a Chilean population over 50

**DOI:** 10.1186/s41155-024-00329-8

**Published:** 2025-01-02

**Authors:** Belén Salinas-Rehbein, Oscar Terán-Mendoza, Vicente Cancino

**Affiliations:** 1 Departamento de Psicología, Facultad de Ciencias de La Salud, Universidad Católica de Temuco, Temuco, Chile; 2 Doctorado en Psicología, Facultad de Educación, Ciencias Sociales y Humanidades, Universidad de La Frontera, Temuco, Chile; 3 Departamento de Psicología. Facultad de Educación, Ciencias Sociales y Humanidades, Universidad de La Frontera, Temuco, Chile

**Keywords:** Social support, Aging, Psychometry, Reliability, Validity

## Abstract

**Background:**

Social support is relevant to studying well-being, quality of life, and health during aging, particularly in people over 50. Therefore, brief instruments that allow its measurement within the clinical evaluation and research processes are necessary. The ENRICH Social Support Scale (ESSI) is a brief and easy-to-use instrument that measures the perception of social support; however, its psychometric properties in people over 50 in the Chilean context have yet to be tested.

**Method:**

This study had a non-experimental, longitudinal panel design in which a sample of 303 people over 50 years of age (*M* = 55.14 years, *SD* = 5.19; 52% women) were administered a survey incorporating sociodemographic variables, the ESSI and the Newsom Social Negativity Scale. 277 participants completed the follow-up survey (*M* = 56.75 years, *SD* = 5.15; 54% women). Confirmatory factor analysis (CFA), structural equation modeling (SEM), invariance analysis, and internal consistency tests were performed to determine the psychometric properties.

**Results:**

The six items from the scale load in a single-factor model obtained an excellent fit to the data and reliability coefficients (α = 0.902; ω = 0.904). The SEM analysis evidenced an inverse correlation between the ESSI and Newsom's social negativity scale, demonstrating evidence of construct validity. Furthermore, in the sex invariance analyses, the scalar level was reached, implying that the items’ meanings are the same for men and women. Finally, the residual level was reached in the temporal invariance analyses, which means that the scale items are consistent in time.

**Conclusions:**

The ESSI is a reliable and valid tool to be used in the Chilean context both in the clinical setting and in research on people over 50. The evidence obtained supports its usability to appropriately measure the perception of social support, which is relevant considering that it has been associated with reports of higher quality of life and lower mortality and morbidity during aging.

## Introduction

Social support, the perception of being loved, valued, and esteemed by members of a social network in which there are mutual obligations (Vila, 2021), is a complex construct that includes the support that people perceive and receive from different sources, such as family, friends, partners, neighbors, or significant others (García-Martín et al., 2016). Social support has various functional aspects: informational support (e.g., providing advice), instrumental or tangible support (e.g., assisting, material aid), and emotional support (e.g., expressing affection) (Menec et al., 2020).

Of these types of support, emotional social support, the perception of being cared for and loved, has received particular attention in research; this involves perceived connection, the feeling of being protected, attraction (in romantic relationships), and positive affect (in non-romantic relationships), these being aspects that favor better coping with stressful situations (Vila, 2021). This has been proved in a longitudinal study with widower older people, in which those who received emotional support consistently for seven years had lower mortality risk compared with people who received instrumental support, which can be attributed to the fact that emotional social support not only plays a role in mitigate loneliness and isolation but also promotes the development of other psychosocial resources, such as self-esteem and self-efficacy (Tzeng et al., 2023).

Health and wellbeing research includes within its areas of interest the identification of elements that increase (risk factors) or decrease (protective factors) the likelihood of an adverse or unpleasant outcome in either physical or mental health. Some widely studied psychological risk factors in aging are loneliness, isolation, chronic stress, and sedentary behavior (Gale et al., 2018; Valtorta et al., 2016; Zhang et al., 2021), while some commonly explored protective factors are, balanced diet (Yannakoulia et al., 2018), physical exercise (Maier et al., 2021) and social support (Gariépy et al., 2016).

Scientific evidence shows that people with high levels of social support have better health outcomes, and a lack of support increases the risk of morbidity and mortality by 53% (Holt-Lunstad et al., 2010). Specifically, social support promotes health behaviors such as physical activity, maintaining a balanced diet, and adherence to medical treatments (Huang et al., 2022). Furthermore, social support is related to less anxiety, depressive symptoms and better sleep quality (Grey et al., 2020), less psychological stress (Lakey & Orehek, 2011), and a lower risk of developing cognitive impairment in older people (Yin et al., 2020).

Social support has been classified as a protective factor across the lifespan, but its impact appears to be even more significant during aging (Shen et al., 2022). That is due, in part, to the fact that a low perception of social support is associated with feelings of loneliness and a negative perception of aging, which can generate emotional responses to stress and anxiety (Czaja et al., 2021; Hawkley & Cacioppo, 2010). Moreover, previous evidence has shown that psychosocial variables related to aging impact people even before they can be considered old because it has been observed that after 50 years old, higher age discrimination is experienced (Ayalon & Cohn-Schwartz, 2022; Ayalon & Gum, 2011). Since social relationship quality predicts better physical and mental health, it is feasible to hypothesize that social support could be a protective factor in aging (Carter et al., 2023).

The protective role of social support on aging is relevant, considering that it is projected that by 2050, one in six people in the world will be 65 years old and over, and 426 million people will be 80 years old and over (United Nations, 2019). In Chile, a South American country, it is projected that by 2050, life expectancy will be 85.4 years and that the percentage of older people will be higher than those under 15 years old (Rojas et al., 2022). In addition, although both physical (e.g., inflammation) and psychosocial (e.g., retirement, decline in social networks) changes may occur at this stage of (Ross et al., 2017; Straka et al., 2021), which could lead to emotional and behavioral responses, in some cases deviations from the norm occur that could lead to pathological states such as depression or cognitive impairment, compromising the person's functionality and independence (Altieri et al., 2021; Park et al., 2014).

Considering that social support is a protective factor for several health outcomes and could promote healthy aging trajectories, it is necessary to have valid and reliable instruments to measure the construct precisely. The most widely used instrument in Chilean studies with older people is The Multidimensional Scale of Perceived Social Support (MSPSS) (Alfonso Figueroa et al., 2016; Vivaldi & Barra, 2012), composed of 12 items that measure the perception of social support from friends, family, and significant others. The analysis of its psychometric properties in older Chilean people was conducted by Arechabala and Miranda (2002) in a sample of 76 participants, obtaining a factorial structure of two factors (family and friends) with an internal consistency measured through Cronbach's alpha (α) of 0.86. In another study by Pinto et al. (2014), a three-factor structure was obtained with 87 older people (family, friends, and significant others) with an internal consistency of α = 0.86. There are at least two concerning aspects regarding the use of the MSPSS in older Chilean people; first, the psychometric properties of the scale have been tested with sample sizes below the recommended in the literature for adequate test power in factor analyses (Goretzko et al., 2021). Second, the measurements have focused primarily on the sources of social support rather than the types of support (informational, instrumental, and emotional).

Gallardo-Peralta et al. (2021) recently adapted the Questionnaire on Perceived Social Support in Chile for a sample of 800 older people. This instrument comprises nine questions on three factors (emotional, informational, and instrumental support) and has an internal consistency of α = 0.92. Although this instrument has adequate psychometric properties, the format requires applying the questionnaire for each source of support explored, which means extending the evaluation time depending on the number of sources of support to evaluate.

A brief alternative for the measurement of social support is the ENRICHD Social Support Instrument (ESSI), initially developed for the Enhancing Recovery Heart Disease (ENRICHD) study to measure the perception of social support in people recovering from ischemic heart disease (Mitchell et al., 2003). This scale is composed of 7 items that measure the frequency with which people experience emotional, instrumental, and informational social support. The ESSI has a frequency scale with five options ranging from 1 (Never) to 5 (Always) for the first six items, whereas item 7 asks about marital status, with a dichotomous response format (Yes/No). In the original study, the scale showed a unifactorial structure and adequate internal consistency (α = 0.95).

Subsequent studies have exclusively utilized items corresponding to the interval measure of the instrument, based on the premise that item 7 targets a structural component rather than a qualitative one, which is an essential element in measuring perceived social support. This six-item structure was used in the study of Cancino et al. (2018), obtaining an internal consistency of α = 0.73; however, due to the nature of the study, they did not analyze the factorial structure or other psychometric properties. The only validation of the ESSI in the Chilean population was conducted by Ortiz et al. (2022) in university students and young and middle-aged adults, obtaining a unidimensional structure of six items, total scalar invariance between men and women with an internal consistency of 0.90 estimated through McDonald's omega (ω). Similar results were found in the study of Gottvall et al. (2019); partial scalar invariance between men and women was reached, with differences only in item two, where the sentence’s meaning was different depending on sex.

Even though the study of Ortiz et al. (2022) obtained excellent psychometric properties; it is worth noting that it did not consider older people as part of its sample; hence, it is impossible to affirm that the ESSI is a valid instrument for older Chilean people. On the other hand, there are sources of validity poorly explored in previous studies with the ESSI in Chile; for example, in the broad spectrum of social relationships, there are other phenomena that also impact health and well-being, such as social negativity, which although it is not the opposite of social support (Villarroel & Ortiz, 2019), the measures should be differentiable from each other. Furthermore, social support is a relatively stable construct over time, therefore, it is necessary to have evidence of temporal invariance (Ortiz et al., 2022).

In synthesis, considering the changes that occur during aging and that social support is a relevant variable given its protective role against psychosocial vulnerability mechanisms and as a predictor of health outcomes, this study aimed to determine the psychometric properties of the ESSI in a sample of people over 50 years old living in Chile. The specific aims were (a) to determine the factor structure of the ESSI, (b) to obtain evidence of temporal and sex invariance, and (c) to analyze the association of the ESSI with a social negativity instrument.

## Methods

### Study design and participants

This study uses data from two longitudinal panel study projects (FONDECYT Posdoctorado 3180534 and FONDECYT Regular 1180463) of adults over 50 years from Temuco, Araucanía region, Chile, which began in 2018. These studies aimed to determine the associations between psychological and physiological variables, obesity, and metabolic syndrome in the general population. The exclusion criteria were being diagnosed with cardiovascular, neurological, or psychiatric disease and under anti-inflammatory treatment, given its possible relationship with the variables of interest of the studies.

We used data from wave 1 (2018), consisting of 303 adults (*M* = 55.14 years, *SD* = 5.19), of whom 52% were women, to test the factor structure of the ESSI, sex invariance, and the association of the instrument with a social negativity measure. To test the temporal invariance of the ESSI, we used data from waves one (2018) and two (2019), comprising data for a total of 277 participants (*M* = 56.75 years, *SD* = 5.15), of whom 54% were women, who completed the follow-up survey in the second year of the study.

### Measures

The ENRICHD Social support instrument (ESSI) (Mitchell et al., 2003; Ortiz et al., 2022). It is a self-report instrument that assesses the perception of social support through seven items; the first six items are answered through a frequency scale with five response options ranging from 1 = "Never" to 5 = "Always." It is a unidimensional measure of the construct. Some item examples are "Someone has been available to listen to me" or "I have been in contact with someone I can trust," in which the higher the score, the greater the perception of social support. For this study, item seven was discarded because of its dichotomous nature. The internal consistency of the scale in the validation study for the Chilean context was ω = 0.903.

Newsom's Social Negativity Scale (Newsom et al., 2005; Ortiz et al., 2017). It is a self-report scale composed of 12 items that measure the frequency with which people experience behaviors they perceive as aversive or unwanted. The response options have a frequency scale ranging from 1 = "Never" to 5 = "Always." The higher the score, the greater the social negativity. Some examples are: In the past month, how often did the people you know "…question or doubt your decisions?" or "…give you unwanted advice?" The Chilean validation study reported a reliability of α = 0.86; for this study, it was α = 0.90.

### Procedure

The Universidad de La Frontera Institutional Review Board evaluated and approved the research protocol. The study participants were contacted via telephone and e-mail. Subsequently, participants attended the university facilities, where research assistants explained the study's aims and procedure details. Those who agreed to participate voluntarily signed the informed consent form. Then, participants completed an online survey in Spanish with sociodemographic, psychological, and behavioral variables of interest. Finally, participants were compensated with $15.000 Chilean pesos ($18 U.S. dollars).

### Data analysis

Preliminary data analysis was conducted to test for multivariate outliers (Mahalanobis distance) and multivariate normal distribution (Mardia's skewness and kurtosis tests). The sample and the ESSI items were characterized using descriptive statistics of central tendency (means) and dispersion (standard deviation). To determine the structural validity of the instrument, Confirmatory Factor Analysis (CFA) was conducted using the Weighted Least Squares (WLS) estimator, given the ordinal nature of the data. Structural equation modeling (SEM) was performed to test the correlation between the latent factors of social support and social negativity to determine construct validity. The social negativity latent factor was modeled with the dimensions of unwanted advice or intrusion, failure to provide help, unsympathetic or insensitive behavior, and rejection or neglect. The adequacy of the model fit was tested through conventional goodness-of-fit indicators, following the recommendations of Ullman and Bentler (2012), who state that a good fit is given by chi-square (χ^2^) not significant (*p* > 0.05), Comparative Fit Index (CFI) ≥ 0. 95, Tucker Lewis index (TLI) ≥ 0.90, (SRMR) ≤ 0.08 and root mean squared error of approximation (RMSEA) ≤ 0.06. Additionally, CFA factor loadings should be above 0.400. Likewise, the reliability was estimated using Cronbach’s Alpha and McDonald's Omega scales with their respective 95% confidence intervals.

Finally, a multigroup SEM analysis was performed following the recommendations of Putnick and Bornstein (2016) to determine whether the scale was invariant by sex and over time. The invariance analysis involves a process of successive restrictions to various parameters in the measurement model to be tested. First, a base model, also known as the same form (configural model), is established in which it is determined that the model fits well in both sets of data. Then factor loadings (metric or weak invariance), intercepts (scalar or strong invariance) and item errors (residual or strict invariance) are progressively constrained to equality. A level of invariance is accepted when the model with more restrictions does not significantly worsen its fit compared to the previous model, which is determined by a decrease in CFI ≥ 0.010 and an increase in RMSEA ≥ 0.015 (Putnick & Bornstein, 2016). The analyses were performed with R Studio software using the *lavaan* package (Rosseel, 2012).

## Results

The preliminary analysis did not reveal the existence of multivariate outliers; however, there was evidence of non-compliance with multivariate normality with significant values for skewness and kurtosis (*p* < 0.001); therefore, a robust variation of the estimator with adjustment of means and variances (WLSMV) was used. Regarding the factorial structure, the single-factor model obtained excellent goodness-of-fit indicators χ^2^ = 18.597(9); *p* = 0.029; CFI = 0.977; TLI = 0.962; RMSEA 0.059 [CI90% = 0.018–0.098]; SRMR = 0.004. The factor loadings were above the established cut-off point (λ = 0.669—0.862) and are presented with the descriptive statistics and bivariate correlations in Table 1. Likewise, the reliability of the scale was excellent in both the first measure (α = 0.902 [95%CI 0.883—0.917]; ω = 0.904 [95%CI 0.879—0.923]) and the second (α = 0.914 [95%CI 0.902- 0.924]; ω = 0.904 [95%CI 0.897- 0.931]).

**Table 1:** Descriptive statistics, factor loadings and correlations between items

				Correlations between items					
	*M*	*SD*	λ	Item 1	Item 2	Item 3	Item 4	Item 5	Item 6
Item 1	4.162	0.824	0.796	-					
Item 2	3.960	0.966	0.831	0.757** [0.705—0.801]	-				
Item 3	4.274	0.921	0.772	0.613** [0.538—0.679]	0.600** [0.523—0.668]	-			
Item 4	3.640	1.039	0.669	0.521** [0.434—0.598]	0.590** [0.511—0.659]	0.477** [0.385—0.560]	-		
Item 5	3.993	1.029	0.862	0.638** [0.566—0.700]	0.676** [0.610—0.733]	0.714** [0.654—0.765]	0.599** [0.521—0.666]	-	
Item 6	4.238	0.981	0.745	0.571** [0.489—0.642]	0.566** [0.484—0.638]	0.631** [0.558—0.694]	0.480** [0.389—0.563]	0.703** [0.641—0.756]	-

*M* Mean, *SD* Standard deviation, λ factor loading; in bold correlation coefficient and in square brackets 95% confidence interval

**p < 0.001

In the multigroup analysis, to determine whether the scale was invariant between men and women, we obtained a configural model (M_0_) with an excellent fit; subsequently, when incorporating the restriction of equality in the factor loadings (M_1_), there was no statistically significant change in the fit. Therefore, we proceeded to establish equality restrictions in the intercepts (M_2_), and it was observed that there was no statistically significant worsening in the model fit; consequently, we tested the level of strict invariance (M_3_) by restricting to equality of the indicators of the items, this last model significantly worsened the fit of the model. Hence, it is not possible to assume this level of strict invariance (Table 2). To test the temporal invariance, the same process of successive restriction of parameters was performed, reaching the highest level of invariance (Table 3).

**Table 2:** Measurement invariance between man and women (*n* = 303)

Model	*χ*^2^ (*df*)	*p*	CFI	TLI	RMSEA (90% CI)	SRMR	Model contrast	ΔCFI	ΔRMSEA
M_0_	25.250 (18)	0.118	0.983	0.971	0.052 (0.000—0.095)	0.038	-	-	-
M_1_	30.029 (23)	0.149	0.983	0.978	0.045 (0.000 – 0.086)	0.047	Δ M1—M0	0.000	-0.007
M_2_	38.639 (28)	0.087	0.974	0.973	0.050 (0.000 – 0.086)	0.052	Δ M2 – M1	-0.009	0.005
M_3_	51.290 (34)	0.029	0.958	0.963	0.058 (0.019—0.089)	0.071	Δ M3 – M2	-0.016	0.008

M_0 _configural model, M1 metric model, M2 scalar model, M3 residual model. χ^2^  Chi square, df  degrees of freedom, CFI  Comparative Fit Index, TLI  Tucker-Lewis Index, RMSEA (90% CI) Root Mean Square Error of Approximation with 90% confidence interval, SRMR Standardized Root Mean Square Residual, ΔCFI Change in Comparative Fit Index, ΔRMSEA Change in Root Mean Square Error of Approximation

**Table 3:** Longitudinal invariance indicators (*n* = 277)

Model	*χ*^2^ (*df*)	*p*	CFI	TLI	RMSEA (90% CI)	SRMR	Model contrast	ΔCFI	ΔRMSEA
M_0_	63.025 (47)	0.059	0.983	0.976	0.035 (0.000—0.056)	0.036	-	-	-
M_1_	58.679 (53)	0.275	0.994	0.992	0.020 (0.000 – 0.044)	0.044	Δ M_1_—M_0_	0.011	-0.015
M_2_	71.735 (59)	0.124	0.986	0.985	0.028 (0.000 – 0.048)	0.049	Δ M_2_ – M_1_	-0.008	0.008
M_3_	66.089 (58)	0.218	0.991	0.990	0.022 (0.000—0.045)	0.047	Δ M_3_ – M_1_	-0.003	0.003

M_0_ configural model, M_1 _metric model, M_2 _scalar model, M_3 _residual model. χ^2 ^Chi square, df  degrees of freedom, CFI Comparative Fit Index, TLI Tucker-Lewis Index, RMSEA (90% CI) = Root Mean Square Error of Approximation with 90% confidence interval; SRMR Standardized Root Mean Square Residual, ΔCFI Change in Comparative Fit Index, ΔRMSEA Change in Root Mean Square Error of Approximation

**Fig. 1:**
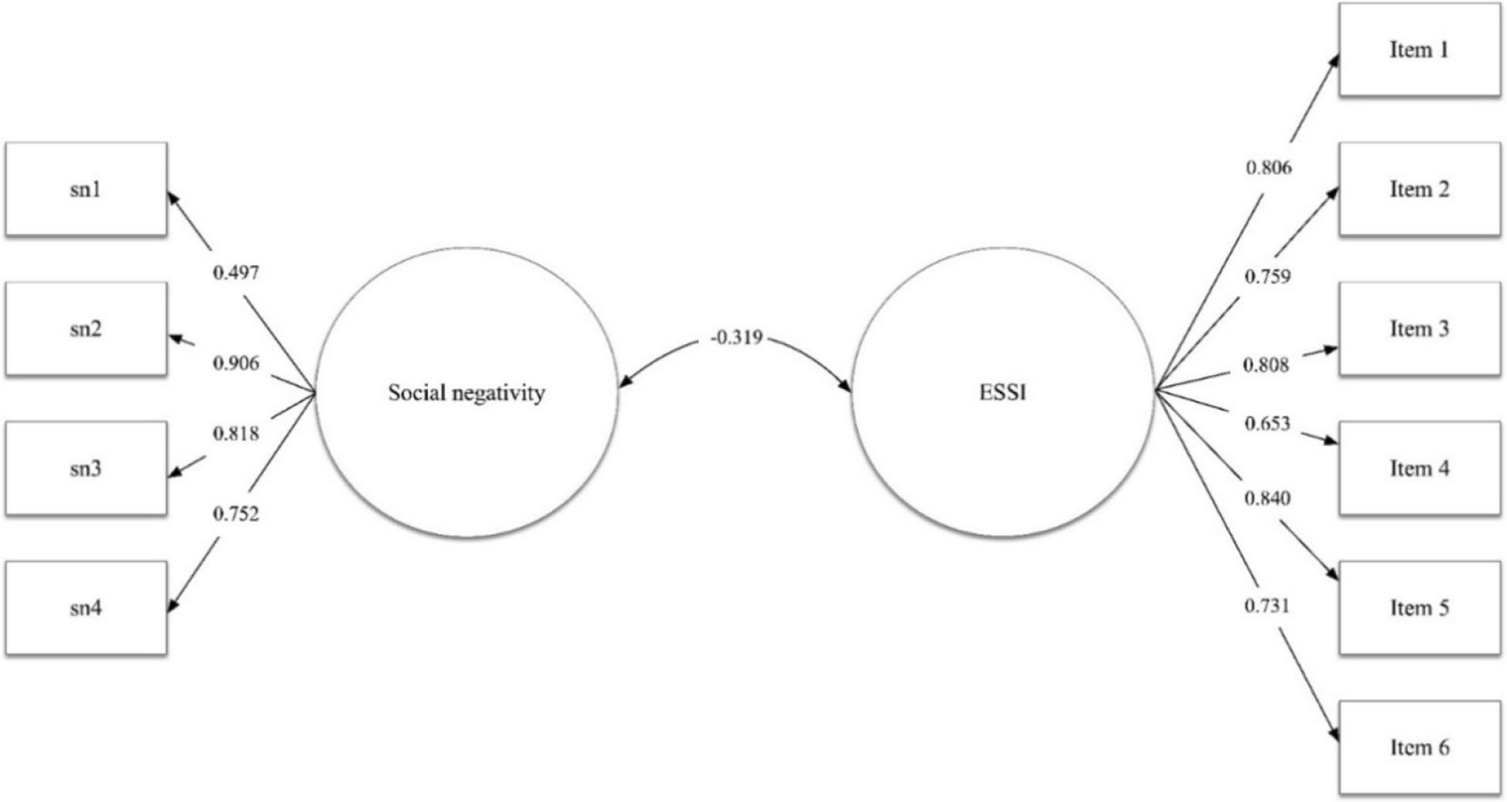
Structural equation modelling for convergent validity

Finally, for convergent validity, the model tested obtained an excellent fit χ^2^ = 44.484 (34);* p* = 0.108; CFI = 0.993; TLI = 0.991; RMSEA 0.032 [CI 90% = 0.000–0.056]; SRMR = 0.061. The covariance between social support and social negativity factors was *r* = 0.319 (Fig. 1).

## Discussion

This study aimed to analyze the psychometric properties of the ESSI in a sample of people over 50 in Chile. The results are consistent with previous studies that identified a unifactorial structure composed of six items (Cancino et al., 2018; Ortiz et al., 2022). The use of the first six questions is relevant for several reasons. The first is that they have an ordinal scale, unlike item seven, which measures marital status in a dichotomous form. Secondly, social relationships are classified into three components: structure, function, and quality; the structure of social relationships accounts for the size of the individual social network and living arrangements (Holt-Lunstad, 2021). Item seven measures a structural component of social relationships but only inquiries about marital status, leaving out all the people who are unmarried but may live with more people or have a large social network. Finally, although previous studies have shown that the three components of social relationships predict health and well-being outcomes, the quality of interpersonal relationships is one of the most important predictors (Asante & Karikari, 2022; Zhaoyang et al., 2019); therefore, it is helpful to have a measure explicitly focused on how interpersonal relationships are perceived, considering that marital status is usually explored in sociodemographic questionnaires and does not directly account for the function of social support.

The scale also showed excellent levels of reliability in both measurements, obtaining an internal consistency similar to that reported in previous studies internationally (Gottvall et al., 2019; Mitchell et al., 2003) and in Chile (Cancino et al., 2018; Ortiz et al., 2022). In addition, this study identified an inverse association of the ESSI with Newsom's social negativity scale. This result is congruent with previous theoretical and empirical evidence (Okun & Lockwood, 2003; Villarroel & Ortiz, 2019). It shows the discriminant validity of the instrument because although both scales are oriented to evaluate interpersonal relationships, they do so in terms of different aspects. Therefore, a significant correlation without a high magnitude is to be expected.

The results confirm that the instrument is invariant at the scalar level between men and women; in other words, the items are understood similarly regardless of sex. Although the residual level was not reached, it is possible to compare the average scores of the variable for both groups, unlike Gottvall et al. (2019), our results achieved full invariance since, at the scalar level, it was unnecessary to release parameters in any of the items, which is consistent with that reported by Ortiz et al. (2022). In addition, the residual level was reached for the analyses of temporal invariance, which reflects that the interpretation of the construct remains stable over time and demonstrates its validity for use in longitudinal design studies.

One of the advantages of the ESSI is that it can be applied briefly, and its correction is simple given its number of items. In addition, the content of the items is easy to understand, with a simple response format, which enables its use in primary care contexts where time management is essential (Hargraves et al., 2017). Considering the implications of social support for health and well-being, it is necessary to have instruments for its measurement within broad research protocols in the social and health sciences, considering that previous evidence reports that people with high social support report better quality of life and show lower rates of mortality and morbidity (Holt-Lunstad et al., 2010; Siedlecki et al., 2014).

Furthermore, considering that psychosocial changes during aging are salient predictors of well-being and health, it is helpful to have valid scales for this population segment considering that the perception of social support varies throughout life and especially in old age, people may experience feelings of loss both in terms of physical functionality, family support networks or friendships and even related to stop carrying out activities such as work; Therefore, contact with others and the perception of support may have a greater relevance as a coping resource in the face of loss than in previous stages in the life span (Wong & Waite, 2016).

This study has some limitations inherent to the use of non-probability sampling that entails considering the characteristics of the sample when attempting to generalize results. Furthermore, self-report measures may be susceptible to social desirability bias, potentially influencing the variability of responses (Vesely & Klöckner, 2020). Therefore, future research could enhance its design by incorporating other social networks' quantifiable measures to contrast with self-report measures. However, it has several strengths: a sample size adjusted to international guidelines for validating psychometric instruments, robust multivariate analysis techniques, and a longitudinal design. Future research could conduct analyses with a larger sample size, making it possible to develop scales; likewise, these findings could be replicated in other contexts culturally different from the Chilean context.

## Conclusion

In conclusion, the results obtained in this study provide a valid and reliable instrument to measure perceived social support in Chilean adults over 50. The ESSI scale has adequate psychometric properties and is invariant over time and between sexes, which allows a comparison between men and women without the differences being attributed to measurement error. Furthermore, the invariance of the scale over time allows the use of the scale for longitudinal studies. This study provides Spanish-speaking professionals and research teams with a valid and reliable instrument to measure perceived social support in adults and older people.

## Data Availability

A clause in the research protocol approved by the Review Board points out restrictions on the availability of these data, so they are not available to the public.

## Abbreviations

CFA: Confirmatory factor analysis
CFI: Comparative Fit Index
ENRICHD: Enhancing Recovery in Coronary Heart Disease
ESSI: ENRICH Social Support Scale
MSPSS: Multidimensional Scale of Perceived Social Support
RMSEA: Root Mean Square Error of Approximation
SEM: Structural Equation Modeling
SRMR: Standardized Root Mean Square Residual
TLI: Tucker-Lewis Index
WLS: Weighted Least Squares
WLSMV: Weighted Least Squares Mean and Variance Adjusted
